# Changes in Japanese Junior High School Students' Sense of Coherence Before and After the Onset of the COVID-19 Pandemic: A Longitudinal Study of Children and Mothers

**DOI:** 10.3389/fpsyt.2021.780443

**Published:** 2022-01-17

**Authors:** Tomoko Omiya, Naoko Deguchi, Yumiko Sakata, Yuriko Takata, Yoshihiko Yamazaki

**Affiliations:** ^1^Division of Health Innovation and Nursing, Department of Public Health Nursing, Faculty of Medicine, University of Tsukuba, Tsukuba, Japan; ^2^Department of Social Welfare, Faculty of Social Welfare, Nihon Fukushi University, Mihama, Japan

**Keywords:** COVID-19, junior high school students, longitudinal study, mothers, sense of coherence, Japanese

## Abstract

We conducted a longitudinal study to clarify the changes in the sense of coherence (SOC); that is, the ability to cope with stress successfully, of 166 Japanese junior high school students and their mothers before and after the onset of the COVID-19 pandemic. First, we analyzed changes in SOC at three time points for all students and divided them into two groups: Group 1 included students with SOC scores that increased or maintained before and after the onset of the pandemic and Group 2 included students with decreased SOC scores after the onset of the pandemic. Second, we conducted a comparative analysis between the two groups. Overall, results indicated that student's SOC scores increased. Additionally, interpersonal stress scores were lower after the onset of the pandemic than before. There were almost no differences in family relationships, financial conditions, or personality tendencies between the two groups. However, Group 2 did not regain their sense of belonging to school. In this group, the frequency of stress experiences in club activities after the onset of the pandemic, troubles with the opposite gender, and inability to catch up with the contents of the subject lecture were high. The accumulation of small stressors may have hindered the maintenance of a sense of school affiliation. Mothers of students in Group 2 either were full-time employees at baseline or had started a new job after the onset of the pandemic. Their children may have been affected by the household's damaged financial budget and changes in mother's working styles. As COVID-19 reduced the number of days students went to school, students' SOC could have reduced had they not felt a sense of presence or belonging due to the lack of participation in club activities, school events, etc. Teachers and mothers should communicate carefully with their students and children, respectively, to develop a sense of belonging.

## Introduction

The Cabinet Secretariat of Japan (1) issued a state of emergency due on 7 April 2020 to COVID-19(Secretariat, 2020), limiting the daily activities of all citizens to develop self-restraint. This included drastic changes to daily life, such as restricting unnecessary activities and allowing going out only for urgent activities, remaining homebound as much as possible, and wearing a mask and washing hands, to which people experienced confusion. On 28 February 2020, the Ministry of Education, Culture, Sports, Science and Technology (MEXT) stated, “From the perspective of preparing for the risk of infection caused daily by many children and teachers gathering for a long time, all schools (elementary, junior high, high, and special needs schools) are requested to be closed all over the country” (2). Consequently, all schools under the jurisdiction of MEXT throughout Japan were temporarily closed. Specifically, 74% of kindergartens, 95% of elementary schools, 95% of junior high schools, 97% of high schools, and 96% of special schools were closed (3). The first state of emergency was lifted nationwide on 25 May. That is, for about 3 months, students were unable to attend school. This was the first time in Japan that all schools were suddenly closed for a long time, and confusion and anguish were reported in the media every day. This unprecedented event of closing the schools may have brought about serious changes among students, but it is unclear what changes occurred.

According to Japanese social customs, the year begins in April and ends in March. March is an important time for the school year ends, with graduation and closing ceremonies normally taking place then. In April, entrance and opening ceremonies are held, and a new academic life starts. School events, rituals, and ceremonies have positive psychological effects, such as motivation, hope, and re-evaluation of experiences (4, 5). However, due to the unprecedented long temporary closure, students were unable to experience these events. Additionally, even if it had been possible to go to school, distributed and staggered school attendance as well as refrain from club activities continued; the current situation was still far from normal school life. No one had experienced this and the drastic changes in daily life have had a great impact on the students. For example, the number of suicides among elementary, junior high, and high school students in Japan in 2020 increased by 100 (25.1%) from the previous year to 499 (6). The MEXT suggests that “social anxiety due to the spread of COVID-19 may have caused this” (6). However, the number of suicides is said to be the tip of the iceberg. Many children may have potentially been distressed, but reports on their situation and how the unprecedented long temporary leave has affected them are limited. There is an urgent need to understand the children' current situation, identify children in extremely unstable situations, and promptly provide care and support to them.

Junior high school students in Japan are in the early stages of puberty, a period referred to as “thunder and storm” (7). In this extremely unstable stage of life, mental and physical growth is remarkable. Considering that suicide has been the leading cause of death among teenagers and individuals in their twenties for many years in Japan (8), it has been said that it is necessary to enhance the “zest for life” among adolescents. Several studies regarding adolescent suicide prevention and strengthening ability to overcome difficulties have been conducted (9, 10). In these studies, “zest for life” has been conceptualized as a sense of coherence (SOC), which is the basic concept of Antonovsky's (11) salutogenesis theory. SOC is described by Antonovsky as the ability to cope well with stress and protect one's physical and mental health even in extremely stressful conditions, leading to healthy growth and development. Adolescent SOC is also said to predict adult SOC, and SOC is extremely useful as an index to approach adolescents (12). Omiya et al. (10) examined the factors related to the SOC of high school students, and the results showed that school sense of belonging, student and teacher acceptance, good family relationships, and mother's SOC were positive factors for high school SOC. SOC consists of three subscales: manageability, comprehensibility, and meaningfulness (11), but the ongoing pandemic is neither understandable nor manageable in everyday life. Almost all club activities that the students have been working on every day have been canceled and developing a sense of meaning has become difficult. In these circumstances, the SOC of adolescent junior high school students is also affected by the pandemic, but there are no reports on the changes before and after the onset of the pandemic.

In addition to these effects, according to a survey by the Japan Gender Equality Bureau Cabinet Office (13) report, due to the pandemic, about 48% of workers in the metropolitan area shifted to telework, about 70% refrained from eating out at events, and about 70% of teens and people in their 20's increased their internet use time. According to Ishida et al. (14), in Japan, one's social network was not only shrinking face-to-face, but also via email and chat. In addition, it is reported that more than 80% of individuals experienced anxiety about the economic recession and lack of infection prevention supplies and information. Moreover, blue-collar workers experienced high levels of anxiety and women experienced stronger anxiety than men. In Japan, many women are forced to work part-time and as non-regular workers; the Japan Gender Equality Bureau Cabinet Office (13) reported that unemployment and suicide during the pandemic are increasing sharply among women. Such economic impacts may have directly affected households and livelihoods, but it is unclear how economic changes have affected adolescents.

Additionally, there is a call for maintaining social distance everywhere to prevent infection (15), and schools are taking every step to avoid crowding. Reportedly, students are instructed to eat silently at lunchtime every day at school. Traditionally, the Japanese school life is characterized by a strong sympathetic “everyone together” pressure, and in adolescence, children are highly concerned about others' opinion and their mental health, and they often find it difficult to ask for assistance even in times of trouble (16). Now that the physical distance from others has increased drastically, it is possible that the psychological sense of distance with friends is also changing, and that it may be working positively or negatively for them. Particularly, living with a mask every day is likely to make it difficult to read facial expressions and understand emotions (17). That is, students' current school and family life is completely different from the lives they had prior to the pandemic. Under these circumstances, it is necessary to clarify the changes in the “zest for life,” or SOC, of junior high school students amid their vulnerable adolescence. If their SOC is decreasing, understanding how they differ from those whose SOC has increased or maintained is important to provide care and support promptly.

We clarified the changes in the SOC of junior high school students before and after the COVID-19 pandemic, particularly comparing those with decreased SOC with those whose SOC increased or remained consistent. Specifically, we thought that the implications for care and support for the decreased SOC group should be clarified.

## Materials and Methods

### Participants

Students from two public junior high schools (junior high schools A and B) in the Tokyo metropolitan area consented to participate in the study with their parents (mainly mothers). With the cooperation of the first grade of junior high school A and the first and second grade of junior high school B, the questionnaire was distributed to students who consented to the survey. Data was collected at three separate time points. The survey acquired the baseline data in the spring of 2019 (March–April), then in the winter of 2019 (October–December), and finally in the summer of 2020 (July–September). The third data collection was after the onset of the pandemic. The parents responded to a baseline and the third survey. Parents were matched with children and 166 pairs were surveyed, from which parent–child data were obtained thrice from them. The parental consent rate for participation in this study was 56 and 46% of the total pairs who responded to all three times was.

### Ethical Considerations

As per the ethical guidelines, since the participants were underage, we first explained the outline of the survey to the parents of the junior high school students in writing and distributed the survey form only to the students who signed their consent forms after obtaining handwritten consent from their parents. We explained to the students in writing and confirmed their intentions by setting a check box on the questionnaire as to whether they agreed to participate. We also explained that participation in the survey was voluntary, there was no disadvantage to non-participation, and they could withdraw at any time. Since this was a longitudinal survey, each student was given a 6-digit random personal ID number, which was used to manage the participant's data. For parent–child data matching, a common parent–child number was assigned to each pair in addition to the personal ID number. However, we explained in writing that these ID numbers were not personally identifiable and that anonymity was maintained. Documents that linked the personal ID number and student name were managed by one person in charge of each school and were locked in an archive so that the individual could not be identified from the answers. The student questionnaires were collected from the school, and then distributed to and collected from the parents through the students. I conducted a survey in accordance with the Declaration of Helsinki. Additionally, this study was approved by the medical ethics review board of the University of Tsukuba (approval number: 1,343, approval date 30 January 2019).

### Variables (Measures)

#### Demographic Variables

Students provided information regarding basic attributes such as their gender and grade.

#### Sense of Coherence

We used the Japanese abbreviated version of the SOC scale, comprising 13 items on a five-point scale, the reliability and validity of which have been verified by Togari et al. (18). This version of the scale is widely used in Japan's national sample surveys and studies involving high school students (9, 10). The SOC-13 has three subscales: comprehensibility (five items), manageability (four items), and meaning (four items). The Cronbach's alpha coefficient for SOC was from 0.789 to 0.903, for mothers and for children, for each time.

#### For Students (Common to All Three Surveys)

##### School Membership Scale

We used the Japanese version of the psychological sense of school membership scale, which was developed by American psychologist Goodenow (19) and the reliability and validity of the Japanese version were verified by Togari et al. (20). This scale has been validated for high school students in Japan. The authors of the current study and the junior high school teachers who collaborated on this study reviewed the content of the scale and determined that it could be used for junior high school students. We also contacted the original developers of the Japanese version of the scale, whose sample included high school students. They agreed and permitted the use of the scale on a sample of junior high school students. It consists of three subscales: the sense of acceptance by students (four items), the sense of acceptance by teachers (five items), and the sense of belonging (this comprises four items; it asks students about the degree to which they are proud of their school and their sense of place at school). The responses ranged on a five-point scale, with higher scores indicating better conditions. The Cronbach's alpha for this scale was from 0.788 to 0.891, for each time.

##### Mental Health Inventory; MHI

The MHI was developed by Berwick et al. (21) as a measure for finding mental health status (especially depression) using five items, and Yamazaki et al. (22) modified it in Japanese. The questions were, “I was quite nervous” and “I was so depressed that I couldn't help it” in five stages from “1. *I was always there”* to “5. *I was't at all”* in the past month. The higher the score, the better the mental state. The Cronbach's alpha for this scale was from 0.801 to 0.849 for each time.

##### Physical Symptoms

We asked about eight physical symptoms (headache, abdominal pain, insomnia, palpitation, dizziness, constipation/doarrjea, body pain and irritability) created by Ben-Sira (23). The responses range on a 4-point scale (*always, sometimes, not so much, not at all*), and the higher the score, the better the condition. The Cronbach's alpha for this scale was from 0.822 to 0.911 for each time.

#### Baseline

##### Children's Report on Parenting in Adolescence Scale

Utsumi (24) developed a scale to measure parents' nurturing attitudes from the children's perspective. In other words, this scale reports children's perceptions of their parents' nurturing attitudes. This scale follows Schaefer's (25) theory and has been tested for internal consistency and reliability. We used three subscales: Acceptance (six items consisting of the child's perception of emotional support and warmth toward the parent's nurturing attitude), Psychological Control (six items related to parent's attempts to control behavior), and Monitoring (three items related to parent's tracking and paying attention to the child's whereabouts, activities, and adjustment). For each item, we asked them to respond on a five-point scale ranging from 1 (*Not applicable at all*) to 5 (*Very well applicable*). Cronbach's alphas for this subscale were 0.901, 0.876, and 0.801.

##### Interpersonal Hypersensitivity Tendency

During adolescence, complaints such as “I'm worried about what people think” and “I'm nervous in public” are frequent. Such anxiety manifests itself as school refusal or depression, leading to adolescents being diagnosed with “adjustment disorder” in psychiatry (26). In this study, we used the “interpersonal sensitivity” sub-scale from the “Interpersonal sensitivity/Privileged self-scale” developed by Muranaka et al. (27). The scale comprises two areas: “Sensitivity to evaluation (Cronbach's α = 0.858),” which is being worried about others' evaluation (nine items), “Excessive reaction to evaluation (Cronbach's α = 0.814),” which consists of items such as being sensitive to other people's words and being shocked by trivial words (five items). The responses of each of these items were based on a five-point Likert scale.

##### Recognition of Stress Experience

We used the “High School Student Life-Related Stress Scale” developed by Ishida et al. (28) and confirmed its reliability and validity for adolescent student life-related stress perception. The developers approved the adaptation of the scale for junior high school students. This scale consists of five domains: (1) teachers, (2) club activities, (3) academic performances, (4) friends and opposite gender, and (5) families. Each domain includes four to five items with a total of 19 items across all domains. Participants respond on a 4-point rating scale (1 = never experienced stress, 2 = occasionally experienced stress, 3 = experienced stress, and 4 = frequently experienced stress). We asked about negative perceptions about school life, such as “I was ignored by my classmates and felt unpleasant.” Additionally, based on discussions among the authors of the present study and previous research (29), we included five stressor items related to club activities, as they are highly significant for Japanese junior high school students. Therefore, we used the adapted version of this scale comprising 24 items on a 4-point rating scale.

#### Mothers

##### Baseline

We asked about the subjective economic condition of the home using a five-point Likert scale from “1. *poor*” to “5. *rich*.” The higher the score, the wealthier they were. Regarding working conditions, we asked them to choose from one of the three options, “working in regular employment,” “working in non-regular employment,” and “not working.”

##### The Parental Attitude Scales

We used a scale developed by Kato et al. (30) whose reliability and validity in measuring the parenting attitudes of parents with children in a wide range of developmental stages from early childhood to late adolescence is verified. It comprises ten items of “acceptance/child-centered” (e.g., paying close attention to children), seven items of “inconsistent indecisive discipline” (e.g., changing the rules made for children), and eight items of “control” (e.g., I want my child to do what I want). It is rated on a scale of five from “1- *Almost not applicable*” to “5- *Very applicable*.” Cronbach alpha was 0.815.

##### Life Events

With reference to the life events concept proposed by Natsume and Murata (31), 20 stressful life events were extracted through discussions among researchers. The questions asked, for example, “Have you experienced the following since the last survey?” and enquired on whether “the company [the participant] worked for went bankrupt,” they “got divorced,” or their “income decreased.”

### Analysis

Simple aggregation was performed for the basic attributes. Regarding the SOC scores of the students, the average scores at the three-time points—spring of 2019 (baseline), winter of 2019, and summer of 2020 (after the onset of the pandemic)—were compared and analyzed by one-way analysis of variance (Bonferroni method). In addition to the total score, the three subscales were analyzed in the same way. A paired *t*-test was performed on the recognition of stress experience scores in winter of 2019 and summer of 2020 to examine whether there was a difference in the perception of stress experience (for teachers, club activities, academic performance, friends, family relationships) before and after the onset of the pandemic.

The 166 students were divided into two groups based on their SOC scores: the increased/maintained group and the decreased group. For the division of the two groups, the total SOC score at the third data collection time point (summer of 2020) was subtracted from the total SOC score at baseline (spring of 2019). If the result of the subtraction was positive or zero, the group was classified as “Group 1,” and if it was negative, the group was classified as “Group 2.” There were 6 people who scored 0 (no change), which was 3.6% of the total. The average SOC score of these six students was 44.3, which was higher than the average, so we concluded that they had maintained a good status before and during the pandemic. Therefore, these six students were classified as Group 1.

Subsequently, we compared the SOC scores of Groups 1 (59.6%) and 2 (40.4%) for spring of 2019 (baseline) and summer of 2020 after the onset of the pandemic, using a *t*-test. The SOC total score, MHI, physical symptoms, and school membership scale (examined by subscale) were then tested at each of two points: spring of 2019 (baseline) and summer of 2020 (after the onset of the pandemic). For mothers, we tested for household economic status and employment status at baseline. After the onset of the pandemic, we tested the proportion of mothers who responded with “yes” or “no” to the 20 items of stressful life event experiences between the two groups. A *t*-test was conducted for continuous variables and a χ^2^ test was conducted for qualitative variables.

In addition, comparisons between the two groups were made using *t*-tests for baseline scores of the Interpersonal hypersensitivity tendency, Children's report on Parenting in Adolescence Scale (examined by subscale), and the Parental Attitude scale. After the onset of the pandemic, in the summer of 2020, comparisons were made between the two groups for scores of the school membership scale by subscales, and for the recognition of stress experience by five domains, including teachers and club activities.

A generalized estimating equation (32) was used as a model to estimate the effect on the mean of the population outcome (population-averaged model). We used the generalized estimating equation-logistic model (GEE-L) to construct a binomial model with the dependent variable set to 0 for SOC decreased group and 1 for SOC increased/maintained group. In the first model (**Table 4**), the baseline values (spring of 2019) of the school membership scale, interpersonal hypersensitivity tendency, Children's report on parenting in Adolescence scale, and the Parental Attitude scale were entered. In the second model (**Table 5**), scores of the school membership scale and recognition of stress experiences, which they responded to in the summer of 2020, were included in each of the sub-domains.

To obtain more specific suggestions, student's recognition of stress experiences was compared between the two groups for each item using *t*-tests. Differences were considered significant at p < 0.05. In general, a significance of *p* < 0.05 is widely accepted, but in exploratory analyses, a relatively large significance level such as 0.1 is sometimes set (33). Since this data deals with very exploratory content, we wanted to mention the significance tendency of 0.1. SPSS Ver. 27.0 was used for statistical analysis.

## Results

Table 1 shows the attributes and characteristics of the 166 research participants. Among the students, 52.4% (87) were female and 47.6% (79) were male. The average age of the mothers was 44.9 years ± 4.2, with the largest number of non-regular workers accounting for 53.6%. Regarding the economic situation of households, most reported an average economic situation (47.6%).

**Table 1:** Attributes and characteristics of subjects (Baseline).

	***n***	**(%) /SD**
**Students**	**166**	
2019 1st grade (12–13 years old) Male	24	(52.2)
Female	22	(47.8)
2019 2nd grade (13–14 years old) Male	54	(45.0)
Female	66	(55.0)
**Parents (Mother)**	**166**	
Age, average, SD	44.9	4.2
**Employment**		
Regular employment	43	(25.9)
Non-regular (part-time job)	89	(53.6)
Unemployed	34	(20.5)
**Marital status**		
Divorce / bereavement	17	(10.2)
Married	149	(89.8)
**Subjective economic conditions**		
Poor / very poor	17	(10.2)
Average	79	(47.6)
Relatively rich, rich	70	(42.2)

**Figure 1:**
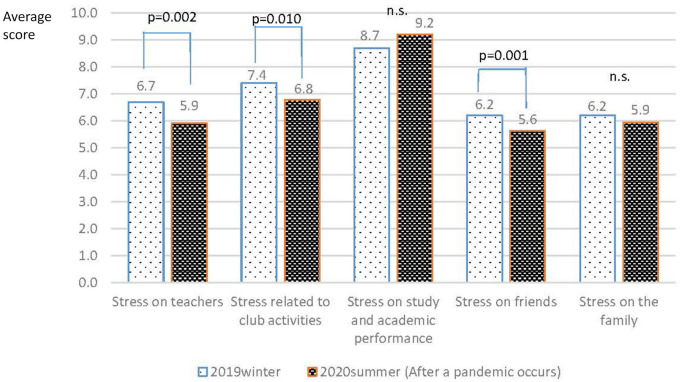
Comparison of student's stress experiences before and after the onset of COVID-19 pandemic.

A comparison of the mean scores of each variable between the baseline and after the onset of the pandemic (Table 2) showed that the SOC scores of all the students increased significantly as a whole after the onset of the pandemic (*p* = 0.006). As for the subscales, scores from the third data collection time point (post-pandemic) were significantly higher than scores at the baseline and second data collection time point (winter of 2019) for both comprehensibility and manageability (*p* < 0.001; *p* = 0.020). Regarding stress experiences at school and home, stress related to teachers (*p* = 0.002), club activities (*p* = 0.010), and friends (*p* = 0.001) were significantly lower after the onset of the pandemic than before (Figure 1). Among the students, 59.6% (99) had maintained or improved SOC scores (Group 1) while 40.4% (67) had decreased SOC scores (Group 2). A comparison of these two groups indicated that Group 2 had higher SOC scores at baseline (*p* = 0.002), but reversed scores in the second (winter of 2019) and third surveys (after the onset of pandemic), indicating a wide difference (Table 3).

**Table 2:**
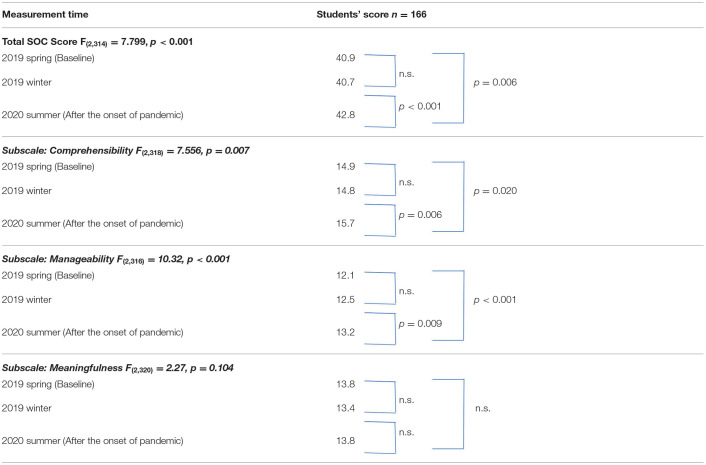
Changes in student's SOC score at 3 points (From spring of 2019 to summer of 2020).

*SOC, sense of coherence. One-factor analysis of variance (repetitive measurement) by Bonferroni method*.

**Table 3:** Comparison of mean scores of variables between two SOC groups of students and mothers before (2019 spring) and after the onset of the pandemic (2020 summer).

**Variables**	**Group 1** ***n* = 99**	**Group 2** ***n* = 67**	***p*-value**
**Student's Variables**			
***Sense of Coherence*** **(range 13–65)**			
2019 spring, t_(164)_ = 3.093	39.6	42.8	0.002
2020 summer, t_(164)_ = −0.834	46.2	37.7	<0.001
***MHI (Mental Health Inventory)*** **(range 5–25)**			
2019 spring, t_(163)_ = -0.413	18.8	18.9	0.949
2020 summer, t_(164)_ = −5.225	20.2	17.3	<0.001
***Physical symptoms*** **(range 8–32)**			
2019 spring, t_(163)_ = -0.413	24.7	24.3	0.680
2020 summer, t_(161)_ = −2.237	25.0	23.2	0.027
**Mothers' Variables**			
***Baseline variables answered by mothers***			
*Family economic condition (the higher the score, the more money you can afford)* (range 1–5)	3.3	3.2	0.817
***Working condition***			
Working in regular employment *n* (%)	18 (20.7%)	19 (33.9%)	0.078
Non-regular staff / not working *n* (%)	69 (79.3%)	37 (66.1%)	
***Life events experienced by parents in summer of 2020 (after the onset of pandemic)***			
“I got a new job”, Yes *n* (%)	10 (10.3%)	13 (21.0%)	0.062
“I got a new job”, No *n* (%)	87 (89.7%)	49 (79.0%)	

*SOC, Sense of coherence. Group 1 = SOC increased/maintained group, Group 2 = SOC decreased group. A t-test was performed on continuous variables, and a χ-square test was performed on discontinuous variables*.

Table 3 also shows that compared to Group 1, physical symptoms and mental health (MHI) worsened in Group 2 after the onset of the pandemic (*p* = 0.027, *p* < 0.001).

A comparison of parental baseline data between the two groups showed no difference in subjective family economic conditions, but at baseline, mothers in Group 2 (*p* = 0.078) showed a trend of being regular employees. “Furthermore, after the outbreak of the pandemic, the percentage of mothers in Group 2 who answered “yes” to the “I got a new job” item were higher than the percentage of those who answered the same in Group 1 (*p* = 0.062).

Tables 4, 5 show the results of the *t*-test, which revealed the relationship between the two variables, and the generalized estimating equation-logistic model (GEE-L), which examined the differences between the two groups. In Table 4, the variables at baseline (spring of 2019) were entered as the explanatory variables to be examined. Consequently, no significant variables were found in the *t*-test or in the GEE-L.

**Table 4:** Generalized estimating equation and logistic model with baseline measurement variables as explanatory variables (*n* = 166).

**Valuables in 2019 spring** **(Baseline)**	**Bivariate analysis**			**GEE-L**						
	**Mean scores for each group**		***T*-test** ***p*-value**	**Coefficient β**	**Standard error**	**Hypothesis testing**		**Odds ratio**	**95% CI**	
	**Group**	**Score**				**Wald χ^2^**	***p*-value**		
**Variables of students**										
**School membership scale**										
Accepted by students (range 4–20)	Group 1 (1)	16.19	0.391	−0.077	0.095	0.656	0.418	1.080	−0.262	0.109
	Group 2 (0)	16.12								
Accepted by teachers (range 5–25)	Group 1 (1)	18.08	0.347	−0.042	0.063	0.436	0.509	0.959	−0.166	0.082
	Group 2 (0)	16.60								
Sense of belonging (range 4–20)	Group 1 (1)	16.64	0.944	0.036	0.088	0.173	0.678	0.964	−0.136	0.208
	Group 2 (0)	16.60								
**Interpersonal hypersensitivity tendency**										
Sensitivity to evaluation (range 9–45)	Group 1 (1)	28.52	0.465	−0.071	0.040	3.116	0.078	1.074	−0.151	0.008
	Group 2 (0)	27.67								
Excessive reaction to evaluation (range 5–25)	Group 1 (1)	15.88	0.134	0.143	0.066	4.764	0.069	1.154	−0.015	0.271
	Group 2 (0)	14.66								
**Children's report on Parenting attitude**										
Acceptance (range 6–30)	Group 1 (1)	24.15	0.879	−0.026	0.049	0.287	0.592	1.026	−0.123	0.070
	Group 2 (0)	24.28								
Psychological control (range 6–30)	Group 1 (1)	12.74	0.737	−0.021	0.040	0.266	0.606	1.021	−0.098	0.057
	Group 2 (0)	13.04								
Monitoring (range 3–15)	Group 1 (1)	12.37	0.940	0.089	0.112	0.638	0.424	1.093	−0.130	0.309
	Group 2 (0)	12.40								
**Variables of mothers**										
***The parental attitude scale***										
Acceptance/child-centered (range 10–50)	Group 1 (1)	40.61	0.429	0.055	0.045	1.512	0.219	1.057	−0.033	0.143
	Group 2 (0)	39.98								
Control (range 8–40)	Group 1 (1)	21.33	0.743	−0.002	0.037	0.002	0.963	1.002	−0.073	0.070
	Group 2 (0)	21.64								
Inconsistent indecisive discipline (range 7–35)	Group 1 (1)	14.17	0.269	0.030	0.053	0.320	0.571	1.030	−0.074	0.135
	Group 2 (0)	13.46								

*Generalized Estimating Equations - Logistic Model (GEE-L). Group 1 = SOC increased/maintained group, Group 2 = SOC decreased group. In the dependent variable, Group 2 was set to 0 and Group 1 was set to 1. Student baseline variables were entered as explanatory variables*.

**Table 5:** Generalized estimating equation and logistic model with 2020 summer (after the onset of the pandemic) measurement variables as explanatory variables (*n* = 166).

**Valuables in 2020 summer** **(After the onset of the pandemic)**	**Bivariate analysis**			**GEE-L**						
	**Mean scores for each group**		***T*-test *p*-value**	**Coefficient β**	**Standard error**	**Hypothesis testing**		**Odds ratio**	**95%CI**	
	**Group**	**Score**				**Wald χ^2^**	***p*-value**			
**Variables of students**										
**School membership scale**										
Accepted by students (range 4–20)	Group 1 (1)	16.74	0.098	−0.004	0.110	0.001	0.969	1.004	−0.219	0.211
	Group 2 (0)	15.97								
Accepted by teachers (range 5–25)	Group 1 (1)	19.36	0.306	−0.045	0.078	0.329	0.566	0.956	−0.198	0.108
	Group 2 (0)	18.73								
Sense of belonging (range 4–20)	Group 1 (1)	16.79	0.004	0.172	0.072	5.694	0.017	1.188	0.031	0.314
	Group 2 (0)	15.36								
**Stress experience recognition**										
Teachers (range 5–20)	Group 1 (1)	5.75	0.041	−0.021	0.123	0.030	0.862	0.979	−0.262	0.219
	Group 2 (0)	6.42								
Club activities (range 5–20)	Group 1 (1)	6.04	0.001	−0.256	0.090	8.174	0.004	0.774	−0.431	−0.080
	Group 2 (0)	7.71								
Academic performance (range 5–20)	Group 1 (1)	8.72	0.008	−0.031	0.059	0.268	0.604	0.969	−0.146	0.085
	Group 2 (0)	10.08								
Friends and opposite gender (range 5–20)	Group 1 (1)	5.34	0.010	−0.098	0.174	0.319	0.572	1.103	−0.438	0.242
	Group 2 (0)	6.11								
Family relationships (range 4–16)	Group 1 (1)	5.57	0.018	−0.041	0.077	0.282	0.595	1.042	−0.191	0.110
	Group 2 (0)	6.52								

*Generalized Estimating Equations - Logistic Model (GEE-L). Group 1 = SOC increased/maintained group, Group 2 = SOC decreased group. In the dependent variable, Group 2 was set to 0 and Group 1 was set to 1. Student baseline variables were entered as explanatory variables*.

In Table 5, the variables observed in the summer of 2020 (after the onset of the pandemic) were used as explanatory variables. *T*-test showed that the sense of belonging score was significantly lower in the decreased group (*p* = 0.004). In stress experience recognition, the stress experience perception in all the domains—Teachers (*p* = 0.041), cub activities (*p* < 0.001), academic performance (*p* = 0.008), friends relationships (*p* = 0.010), and family relationships (*p* < 0.018)—was higher in the decreased group. In the GEE-L analysis, the decreasing group was set to 0 and the increasing/maintaining group was set to 1. The results showed that the sense of belongings was positively related (β = 0.172) and the odds ratio was 1.188 (*p* = 0.017). In terms of stress experience, club activities had a negative relationship (β = −0.256) and the odds ratio was 0.774 (p = 0.004).

As a result of examining the difference in the frequency of stress experience between Groups 1 and 2 for each specific item, in Table 6, five items related to club activities (*p* < 0.001–0.026), two items related to academics (*p* = 0.020), two items related to friends and gender relationships (*p* = 0.020, 0.040), and one item related to family relationships (*p* = 0.011), showed high experience frequency recognition in Group 2.

**Table 6:** Comparison between two groups regarding the mean score of stress experience recognition.

**Details of stress experience (question items)**	**Group 1** ***n*** **=** **99**		**Group 2** ***n*** **=** **67**		**Significant difference in two groups *p*-value**
	**Mean score**	**(SD)**	**Mean score**	**(SD)**	
**Club activities**					
*I couldn't keep up with the practice of club activities:* t_(104.0)_ = 2.264	1.0	(0.33)	1.2	(0.49)	0.026
*The practice of club activities was tough:* t_(96.2)_ = 2.630	1.2	(0.50)	1.5	(0.83)	0.010
*It was difficult to balance club activities and study:* t_(97.3)_ = 3.622	1.4	(0.72)	2.0	(1.18)	<0.001
*I made a mistake in club activities and was scolded:* t_(86.2)_ = 2.454	1.1	(0.40)	1.4	(0.82)	0.016
*No matter how much I practice in club activities, I'm not good at it*: t_(112.2)_ = 3.017	1.2	(0.57)	1.6	(0.77)	0.003
**Academic performance**					
*I couldn't understand the content of the lesson or the teacher's explanation:* t_(102.1)_ = 3.039	1.4	(0.72)	1.9	(1.01)	0.020
*I couldn't answer the teacher's question during class:* t_(163)_ = 2.353	1.4	(0.76)	1.7	(0.86)	0.020
**Relationship with friends and the different gender**					
*I was made fun of regarding my face and body style:* t_(81.7)_ = 2.377	1.1	(0.37)	1.4	(0.82)	0.020
*I am disliked by the opposite gender:* t_(79.5)_ = 2.089	1.0	(0.26)	1.2	(0.63)	0.040
**Family relationships**					
*My parents told me to study more:* *t*_(112.0)_ = 2.575	1.5	(0.81)	1.9	(1.09)	0.011

*Only the question items with significant differences are shown. Group 1 = SOC increased/maintained group, Group 2 = SOC decreased group. Mean score for each group was calculated with never experienced = 1, occasionally experienced = 2, experienced = 3 points, and frequently experienced 4 points*.

## Discussion

### Students' SOC Scores

The SOC scores of the junior high school students improved unexpectedly. Losing direct contact with non-family members and the sudden interruption of the normal “going to school” routine due to the nationwide lockdown may have reminded them of the happiness of being able to go to school. This is similar to when the residents of Japan experienced the shock of “suddenly changing their ordinary lives 1 day at a time” when an unprecedented earthquake called the Great East Japan Earthquake struck Japan in 2011. During that time, people also realized how dear everyday life was (34). In the current case, experiencing the pandemic, school closures, and resumption of school due to COVID-19 may have reaffirmed the joy of going to school every day' among the students. Moreover, the reopening of schools (4), which is a sign of order, may have presented a hope for the future.

SOC is nurtured by family habits in which daily routines are habitually repeated, such as daily greetings and regular declarations such as “I'm leaving!” and “I'm home!” between family members (35). Previous studies have pointed out that stable habits provide good foreseeing and a sense of security, while anxiety reduces SOC (9, 36). Face-to-face communication with peers and homeroom teachers in the same class at school gives students a sense of security and allows them to evaluate their future from regular timetables and fixed annual events. This was also reflected by the significantly higher comprehensibility scores in SOC than in the baseline. Even though the virus was previously unknown, it is now gradually becoming a known (comprehensible) phenomenon accompanied by the feeling that students can go back to school.

Antonovsky (11) also states that fostering manageability is a load-balanced experience that is neither overloaded nor underloaded. According to Feldt et al. (37), doing various tasks and homework required at school means gaining proper experience in balancing the load, and that successful experience leads to strengthening SOC. With the reopening of schools, students may have regained an environment that fosters manageability through daily experiences such as completing daily homework and tasks at school. Alternatively, the fact that only the meaningfulness subscale scores did not increase may indicate that the COVID-19 pandemic has not reached a sense of significance and value in every student's life and daily living yet.

Overall, the frequency of the recognition of interpersonal stress experiences after the onset of the pandemic, especially regarding teachers, club activities, and friends, which are closely related to school, were significantly reduced. This may be because the pandemic has reduced physical and social interaction with people. Thus, for some adolescents that are sensitive to interpersonal relationships or having difficulty being in relationships, the pandemic may have worked in their favor.

### Two-Group Comparison of Students' SOC Scores

The results of this study showed that 40.4% of students had below-baseline SOC scores and that they were unable to recover from their decreased SOC. A comparison between Groups 1 and 2 showed that there was almost no difference in the original family rearing attitude and personality traits of the child, whilst showing a difference in the experience at school after the onset of the pandemic. This indicates the impact of school life on adolescents. Adolescents live most of their days at school and gain much of their adolescent “life experiences” there (38). Therefore, school-related experiences may be a more crucial factor in the formation and development of SOC during adolescence than home or community experiences. Furthermore, the impact of school life may have become relatively large because of the pandemic, limiting social life. Schools and teachers need to assess student's time spent at school carefully, their facial expressions, behavior, and relationships with their peers. Particularly, Group 2 had more physical symptoms such as physical and mental disorders than Group 1. The frequency of going to the infirmary and participation status in physical education classes at school, as well as whether the child is sleeping well at home are considered checkpoints, and it is possible to detect a reduced will to live in students and reduced vitality from complaints related to poor physical condition.

Characteristically, in Group 2, the sense of belonging remained low and did not improve on the school membership scale, showing that students find it more challenging to feel comfortable and attached to the school than to be recognized by teachers and other students (friends) (39). Although the school closure has been lifted, the shortened lessons continue and with this shortened time of being at school physically, some children may be still be unable to develop “feelings of having one's own place at school.” Even though there are restrictions, it is necessary to devise ways to enhance each student's sense of belonging during school events.

For households, there was no significant difference in baseline subjective economic conditions, but there were more full-time mothers in Group 2 and more mothers started new jobs after the outbreak of the pandemic. This may possibly be because families found themselves in economic situations that prompted mothers to seek full-time employment or new jobs to meet household financial needs and supplement the household budget. The pandemic has had a major impact on the global economic situation (40) and the aftermath extended to families with junior high school students, seemingly affecting their mental health.

When the two groups were compared on the items of stressful events, the results showed that Group 2 experienced more stress than Group 1, and the stress experience in club activities was the highest among the five items. Club activities in Japanese junior high schools are extracurricular programs that students spend a lot of time on after school and on weekends, and they vary from physical activities, such as baseball, soccer, and volleyball, to cultural activities, such as chemistry and theater. It is often obligatory for everyone to participate in one of the club activities according to their interests (41). Additionally, club activities are outside the curriculum and are composed of students who wish to actively participate in them beyond their grades and classes (42).

According to Finnish public health scholars, Konu and Rimpelä (43), psychosocial well-being in adolescents includes not only physical health but also successful coping with developmental tasks. They reported that it can be classified into four categories: school conditions (having), social relationships (loving), means for self-fulfillment (being), and health status. Club activities have been shown to contribute greatly to self-fulfillment (being). Self-fulfillment is a successful response to problems related to self-esteem, competence, and self-determination. Improving abilities through steady efforts in cultural and sports activities and achieving good results means increasing self-esteem and achieving self-fulfillment.

Sumiya and Muto (42) demonstrated that club activities increase satisfaction with school life for students with low-class satisfaction. Due to the influence of the pandemic, many major events such as cultural festivals, athletic meets, and choral festivals were canceled in Japanese junior high schools. For students, self-fulfillment in school life other than school grades may have been the last bastion of club activities. Small setbacks, annoyances, and difficult experiences in club activities can impede school adaptation, sense of belonging, and SOC recovery. Schoolteachers need to understand that the reduction in school events during a pandemic means that each of the few barely viable school events, club activities, and classroom activities will have relatively greater significance to students. Teachers should pay close attention to the activities and reactions of students. It is important to talk to students to enhance their sense of belonging and devise a mechanism to obtain a small sense of accomplishment.

### Limitations of This Study

First, the results of the study are limited to two junior high schools. Both were public schools; one was an integrated school (including both junior high and high school) with an entrance examination, and the other was a junior high school without an entrance examination. We chose different types of junior high schools to consider diversity as much as possible, but we analyzed only 166 students and mothers. It is possible that the number of Covid-19 PCR-positive patients in the region was different, or that the results differed between local cities and rural areas.

Care must be taken when generalizing the unexpected increase in SOC scores after the onset of the pandemic. According to Antonovsky (11), SOC in adulthood is said to increase slowly with age, but very few studies have investigated within-year or multi-year variations in adolescents. In Japan, a longitudinal study of high school students (38) reported that SOC decreased in both males and females from the first to the second year and that it may be maintained or decreased from winter to spring and from spring to summer. Therefore, it may be difficult to believe that the rise in this report is age- or season-related. Diverse longitudinal studies in early adolescents are needed in the future to pursue the relationship between this rise and the pandemic. In addition, this study only reports data for 166 students and mothers from two schools. More diverse and more junior high school verifications are required.

Additionally, the situation may be more serious in households who did not agree to participate in the survey from the beginning. It is necessary to carefully observe and care for complaints of physical and mental disorders not only in the decreased SOC group but also in families and their children (students) who did not consent to the survey.

## Conclusions

We analyzed 166 parent–child pairs for changes in the SOC of junior high school students and their mothers before and after the onset of the pandemic. Overall, the SOC scores of junior high school students after the onset of the pandemic were higher than those before the outbreak, but about 40% of the student's scores did not recover. When comparing the increased/maintained SOC group with the decreased SOC group, there was no significant difference in baseline data between the two groups. The decreased SOC group may have not recovered their sense of belonging to school after the onset of the pandemic and there may be a combination of troubles in friendships at school and negative experiences in club activities. Since the decreased SOC group often complained of physical difficulties, it is important for teachers and families to listen carefully to the complaints of students, clarify the cause of the upset, and take care of it. Additionally, even with the restrictions imposed by COVID-19, it is necessary to take measures such as holding school events to the extent possible and calling out to each student to create a place for students and expand their playing field. The COVID-19 pandemic is ongoing worldwide. As long-term effects of the pandemic are expected, attention and care for vulnerable adolescents are crucial. In the future, it will be necessary to verify the effects of the pandemic over the long term. Even if it does not seem to have any effect now, a prolonged pandemic may have a different effect. Moreover, there is a possibility that the consequences of the effects will be completely different after 1 or 2 years; therefore, continued research is required. Additionally, students and mothers who declined to participate in the survey may be facing more serious problems, so it may be important to make provisions for support and care for these individuals.

## Data Availability Statement

The datasets presented in this article are not readily available because the data in this survey is a parent-child survey of adolescent junior high school students and is highly confidential, including information on personality trends and financial conditions associated with legal minors. Schools surveyed do not allow disclosure of raw data, even anonymously. Requests to access the datasets should be directed to Tomoko Omiya, toomiya-tky@umin.ac.jp.

## Ethics Statement

The studies involving human participants were reviewed and approved by the medical ethics review board of the University of Tsukuba. Written informed consent to participate in this study was provided by the participants' legal guardian/next of kin.

## Author Contributions

TO presented the structure, ideas of the research, and wrote the entire paper. YS provided advice on the composition and writing of the treatise. ND and YT coordinated the field and advised as an adolescent expert. YY supported the whole concept of SOC, solidified the theory, and advised the discussion. All authors contributed to the article and approved the submitted version.

## Funding

This study was supported by JSPS KAKENHI Grant Numbers 17K01798 and 21K11077.

## Conflict of Interest

The authors declare that the research was conducted in the absence of any commercial or financial relationships that could be construed as a potential conflict of interest.

## Publisher's Note

All claims expressed in this article are solely those of the authors and do not necessarily represent those of their affiliated organizations, or those of the publisher, the editors and the reviewers. Any product that may be evaluated in this article, or claim that may be made by its manufacturer, is not guaranteed or endorsed by the publisher.
